# Evaluating the feasibility of prolonged-release buprenorphine formulations as an alternative to daily opioid agonist therapy regardless of prior treatment adherence: a pilot study

**DOI:** 10.1186/s40814-023-01348-5

**Published:** 2023-07-04

**Authors:** Bernadette Hard, Mohan DeSilva

**Affiliations:** Kaleidoscope Drug Project, Resolven House, St Mellons Business Park, Fortran Rd Cardiff, Wales, CF3 0EY UK

**Keywords:** Prolonged-release buprenorphine, Opioid-use disorder, Opioid substitution therapy, Opioid agonist therapy, Psychosocial, Adherence, Long-acting buprenorphine

## Abstract

**Background:**

Effective opioid agonist therapy (OAT) depends on good patient adherence. However, the daily, supervised administration of standard OAT represents a significant burden to patients and often drives poor adherence. Prolonged-release buprenorphine (PRB) formulations may mitigate some of this burden, enabling clinic visits to be substantially reduced. For treatment guidelines to be effective, the likely benefit of a transition to PRB therapy in different patient populations must be established.

**Methods:**

The aim was to determine the feasibility of assessing PRB as an alternative to daily OAT in two groups: those currently adhering well to daily OAT (group 1, *N* = 5) and those not currently showing adherence or a positive response to daily OAT (group 2, *N* = 10). This open-label, prospective, non-controlled pilot study was conducted at the Kaleidoscope Drug Project in South Wales, UK. Participants were assessed for history, drug use, psychosocial assessment scores, and clinical severity at baseline and after 6 months of treatment. Primary outcomes were the feasibility of assessing PRB as an alternative to daily OAT and the acceptability of PRB therapy in each group. Secondary outcomes were treatment response, on-top drug use, psychosocial measures, and assessment of clinical severity.

**Results:**

Participants from both groups demonstrated high levels of participation with assessment protocols at both baseline and 6-month follow-up, indicating study feasibility. PRB treatment was acceptable to the majority of participants, with all of group 1 and 70% of group 2 adhering to PRB therapy for the duration of the study and opting to persist with PRB therapy over other OAT options after study completion. All participants who remained on treatment demonstrated marked improvements in psychosocial and clinical severity assessment scores, with some returning to employment or education. On-top drug use remained absent in group 1 and was reduced in group 2.

**Conclusions:**

Evaluation of transition of participants from daily OAT to PRB therapy was shown to be feasible, acceptable, and effective across both groups. A larger randomised controlled trial is warranted, particularly to assess PRB therapy in participants with a history of poor treatment engagement, as the need for therapy is greater in this group and their management is associated with higher costs of care.

## Key messages

- *What uncertainties existed regarding the feasibility?* It is unclear whether participants currently adhering well to daily OAT (group 1) would be willing to transition to and persist with a new form of therapy. Likewise, it was unknown whether participants not currently adhering to daily OAT (group 2) would be willing or able to engage with services to receive weekly or monthly injections. In addition, the feasibility of conducting psychosocial assessments, clinical severity scores, and urine drug screens (UDS) at baseline and 6 months in these two groups was unknown.
- *What are the key feasibility findings?* Of 15 participants recruited to the study, 100% (5/5) of group 1 and 70% (7/10) of group 2 successfully adhered to PRB therapy for 6 months, indicating the dosing regimen was both feasible and acceptable to participants. Collection of psychosocial and clinical severity data at baseline and 6-month follow-up was feasible in both groups. It was not possible to collect UDS data for all participants at follow-up, due to the COVID-19 restrictions in place at the time.
- *What are the implications of the feasibility findings for the design of the main study*? Participants from both groups were able and willing to engage with treatment regimens and assessment protocols. As such, a larger study designed using the same protocols would be feasible. Ease of collection of samples for UDS remains uncertain, due to limitations with sample collection whilst COVID-19 restrictions were in place. Future studies should assess participants across multiple clinics to obtain a more diverse sample.

## Background

Opioid agonist therapy (OAT) forms part of the standard treatment for opioid-use disorder (OUD) and is known to be effective in reducing patient mortality and morbidity, subject to adherence [1]. OAT medications, such as methadone and sublingual buprenorphine, can provide patient stability by reducing on-top drug use, withdrawal symptoms, cravings and needle use [1]. However, many OAT programmes require daily administration, often with mandatory supervision at treatment services, resulting in significant burdens and disadvantages for the patient and impacting upon opportunities to sustain employment and/or education [1–4]. Moreover, daily OAT is commonly associated with significant stigma and discrimination, whilst the risk of diversion and misuse of the prescribed medicine should be considered [3, 5–7].

In recent years, treatment options have expanded and now include a range of prolonged-release buprenorphine (PRB) formulations with weekly, monthly, and 6-monthly dosing preparations available, depending on the country. Such treatments are aimed at mitigating some of the concerns of daily supervised treatment [8]. PRB formulations are known to be as effective as daily OAT, with a comparable tolerability profile, but necessitate considerably less frequent clinic attendance [9, 10]. This has a number of potential benefits for patients, including reduced discrimination and stigma, fewer visits to healthcare settings and improved quality of life, with more time to devote to work, education, family and self-care [2, 9, 11–15]. Moreover, both weekly and monthly subcutaneous buprenorphine formulations have shown high levels of patient satisfaction in open-label follow-up and safety studies [12, 14].

The relevance of prolonged-release formulations increased during the COVID-19 pandemic and nationwide lockdowns. Physical distancing requirements led to reduced access to OAT treatment services as well as to harm reduction and psychosocial interventions [16–19]. In response to this, patients in some countries were provided with longer prescriptions or take-home doses, measures that may have led to ineffective treatment monitoring and, consequently, increased opioid overdoses and fatalities [19, 20]. By contrast, the devolved Welsh government introduced policy changes that enabled treatment services to increase their use of PRB to address some of the safety and practical issues of OUD treatment in the pandemic environment. These changes extended the use of PRB to a wide range of patients and contrasted to previously published guidance from the National Institute for Health and Care Excellence (NICE) in the UK, which had suggested that PRB may be most suited to patients on a stable therapeutic dose of sublingual buprenorphine, or those who may struggle with daily supervised dosing due to work/education or remote location [21].

It is therefore necessary to better understand which patients would be most suited to transition to PRB therapy. It is currently unclear to what degree patients who already adhere well to daily treatment would derive further benefit from PRB therapy and whether patients with a history of poor daily treatment engagement would be good candidates for PRB treatment. As this more complex group is already associated with disproportionately high costs of care, it is particularly important to understand whether PRB would be a cost-effective treatment strategy.

The research questions were:

1. How feasible is this study to assess PRB as an alternative to daily OAT? Objectives:
  i. Measure ability to collect complete datasets for psychosocial and clinical assessments and drug-use data at baseline and 6-month follow-up
2. How acceptable are PRB treatment regimens to participants? Objective:
  i. Calculate the proportion who adhere to treatment
  ii. Calculate the proportion opting to continue with PRB therapy after study completion
3. What is the potential efficacy of PRB therapy? Objective:
  i. Measure changes in scores for clinical and psychosocial wellbeing
  ii. Assess the proportion of participants engaging with on-top drug use

All research questions were assessed in participants successfully adhering to daily treatment (group 1) and in participants unable to adhere to daily treatment (group 2).

## Methods

### Overview

This open-label, prospective, non-controlled pilot study enrolled patients with OUD attending the Kaleidoscope Drug Project in South Wales, UK. Recruitment occurred between July 2019 and January 2020, with most participants recruited during October–December 2019. Assessments were conducted at two timepoints: treatment initiation (baseline) and after 6 months of PRB therapy (follow-up). If patients discontinued treatment prior to 6-month follow-up, data were collected at the point of discontinuation, where possible. Participants were able to continue PRB therapy beyond the 6-month study endpoint, if desired. This pilot study was conducted in compliance with the CONsolidated Standards Of Reporting Trials (CONSORT) statement [22]. The protocol was approved by the Kaleidoscope Clinical Governance Board in accordance with the UK Policy Framework for Health and Social Care Research ethics service. Funding for PRB treatment was provided by the Welsh Government; no other external funding was received. All participants were provided with written information about the treatment and study participation, and written informed consent was obtained.

### Study participants

Consecutive patients were assessed for suitability by prescribing physicians at the Kaleidoscope Drug Project services and offered the opportunity to start OAT with PRB, or to change their existing OAT to PRB if already on treatment. Patients taking > 30 mg daily methadone were excluded due to difficulties associated with transitioning from a high dose of daily medication to weekly/monthly PRB therapy.

Participants were recruited into one of two groups depending on response and current adherence to daily OAT (Table 1). Group 1 included those currently responding and adhering to daily OAT and group 2 included those either not responding or not adhering to daily OAT.

**Table 1:** Defining criteria for groups 1 and 2 at the time of recruitment

Characteristics of group 1	Characteristics of group 2
Fully adherent to daily OAT in the 3-month period prior to study initiation, defined as: • No episodes of treatment drop-out (≥ 3 missed consecutive doses), and • No recorded concerns regarding missed doses by the pharmacy or clinic dosing team AND Responding to daily OAT in the 3-month period prior to study initiation, defined as: • No on-top drug use detected by UDS or self-reporting	Not currently receiving treatment with daily OAT or poor adherence to treatment in the 3-month period prior to study initiation. Poor adherence defined as: • One or more recorded episodes of treatment drop-out (≥ 3 missed consecutive doses), or • Concerns had been recorded by the pharmacy or clinic dosing team regarding missed doses AND/OR Lack of response to daily OAT in the 3-month period prior to study initiation, defined as: • On-top drug use detected by either UDS or self-reporting

Daily OAT includes buprenorphine oral lyophilisate, sublingual buprenorphine or methadone (≤ 30 mg)

### Sample size

No sample size power calculations were required as the main outcomes were feasibility and acceptability.

### Transition to PRB therapy

Participants were transitioned onto treatment with PRB solution for injection (Buvidal, Camurus) under the guidance of a prescribing physician [21]. A rapid titration approach was utilised to transition all participants in group 1 and group 2 onto PRB. The dosing strategy was determined by the treating physician based on clinical judgment and participant preference, with the main aim of providing rapid stabilisation. All participants were given either a single weekly dose (16 mg or 24 mg) before transitioning to monthly doses or were given monthly doses from the outset. Monthly doses were given at 64, 96 or 128 mg of PRB, dependent on individual need. Participants from group 2 not receiving daily OAT at baseline were first administered 4 mg of buprenorphine oral lyophilisate (Espranor, Martindale) and observed for 1 h to confirm the absence of precipitated withdrawal or poor tolerability before they could begin the transition to PRB.

### Outcome measurements

At baseline, all participants completed a semi-structured interview with the attending clinician, which included questions on drug-use history, prior/current OAT use, prior/current response to OAT and engagement in education or employment over the 6-month period prior to study participation. A non-structured interview was also conducted to collect a full history, including past use of drugs and alcohol, housing/homelessness, family and social relationships, education, work, engagement in crime, engagement with social services, other health problems and general concerns. Participants were also asked to provide a urine sample for a UDS during their visit to the clinic which, together with self-reported drug use, was used to determine baseline drug use. Finally, baseline scores for the psychosocial and clinical severity assessments were collected. These assessments included:

- The Clinical Outcome Routine Evaluation 10 (CORE-10) assessment, used to measure common presentations of psychological distress [23]
  - Score categories include ≤ 10 (non-clinical range); 11–14 (mild psychological distress); 15–19 (moderate psychological distress); 20–24 (moderate-to-severe psychological distress); and ≥ 25 (severe psychological distress). This assessment was completed by the clinician, in consultation with the participant.
- The Social Satisfaction Questionnaire (SSQ), used to measure levels of social support available and levels of satisfaction with the social environment [24]
  - Each question was scored as 0 (very dissatisfied), 1 (dissatisfied), 2 (fairly satisfied) or 3 (satisfied), with eight questions in total. Total scores for the SSQ questionnaire therefore range from 0–24. This assessment was completed by the clinician, in consultation with the participant.
- The Clinical Global Impression Severity scale (CGI-S), used to measure the severity of a participant’s illness, based on the OUD International Classification of Diseases (ICD) criteria [25]
  - Scores range from 1–7, with 1 indicating no illness and 7 indicating the participant is extremely ill. This assessment was conducted by the attending clinician. The same clinician conducted this assessment for every participant, both at baseline and 6-month follow-up.

The semi-structured interviews to assess drug use and treatment adherence, the UDS and the psychosocial/clinical severity assessments (CORE-10, SSQ and CGI-S) were repeated when the participants attended the clinic after 6 months of PRB treatment. In addition, participants were given the option to remain on PRB treatment beyond 6 months and asked to provide feedback on their experience of PRB therapy. For those who opted to discontinue treatment prior to the 6-month follow-up, these measures were collected at the point of disengagement, where possible.

### Primary outcomes

#### Feasibility

The feasibility of the study was assessed by the ability to collect complete datasets for the CORE-10, SSQ and CGI-S assessments, and drug-use data (UDS and self-reported), at both baseline and 6-month follow-up appointments.

#### Acceptability

Participant acceptability of the treatment protocol was assessed by:

1. Treatment adherence at 6-month follow-up: the percentage of participants who received their scheduled treatment doses as planned and on time, including the single weekly dose given to some participants as part of the rapid-transition phase. In the case of participants who disengaged from the study prior to the 6-month follow-up, treatment adherence prior to disengagement was considered.
2. Treatment preference: the percentage of participants opting to continue with PRB therapy beyond 6 months over other OAT options, assessed at the 6-month follow-up appointment. In the case of participants who disengaged from the study prior to the 6-month follow-up, feedback on treatment preference was collected at the point of discontinuation, where possible.

### Secondary outcomes

#### Drug use

A UDS was conducted at both baseline and 6-month follow-up visits to the clinic, with urine samples collected on-site. Self-reported drug use, collected during the semi-structured interview, was also assessed at both baseline and 6-month follow-up.

#### Psychosocial and clinical severity evaluation

Psychosocial measures and clinical severity were assessed at both baseline and 6-month follow-up, including the CORE-10 to assess levels of psychological distress, the SSQ to assess satisfaction with available social support, and the CGI-S to assess participant illness severity.

#### Treatment response

Adherence to treatment, improvement in psychosocial scores and level of on-top drug use was used to classify participants as complete responders, partial responders or non-responders (Table 2).

**Table 2:** Treatment response classification criteria

Treatment response	Criteria
Complete response	• Full adherence to treatment schedule • No detected or self-reported on-top drug use
Partial response	• Full adherence to treatment schedule • Some detected or self-reported on-top drug use
No response	• Withdrawal from PRB treatment or missed doses • Some detected or self-reported on-top drug use

#### Return to employment or education

Self-reported levels of employment or engagement with education were assessed using semi-structured interviews at baseline and follow-up. The definition of education included online or face-to-face courses, either part-time or full-time, which resulted in a formal certificate of completion or educational attainment.

### Statistics

This study was not powered to demonstrate differences between groups and no statistics were prespecified. Descriptive statistics including means and standard deviations were calculated for continuous variables. Two-tailed, paired sample *t*-tests were used to determine indicative differences between CGI-S, SSQ and CORE-10 scores pre- and post-treatment within each group. *P* < 0.05 was taken as the level of significance. Scoring systems for each assessment have been previously published [23–25].

## Results

### Demographics and baseline characteristics

A total of 15 participants were recruited (group 1, *n* = 5; group 2, *n* = 10). Demographics and baseline characteristics are shown in Table 3. As per the pre-defined group criteria, all participants from group 1 were fully adherent to daily OAT prior to PRB initiation and had no on-top drug use as assessed by UDS and self-reporting. Nine participants from group 2 were not receiving any OAT at baseline, while one taking daily methadone reported poor response and continued on-top drug use. Of the nine participants in group 2 with available treatment data, the mean percentage of days spent on treatment in a period of 3 to 6 months prior to PRB initiation was 27% (SD, 23%; min–max range, 0–58%), indicating poor adherence to daily OAT among this group.

**Table 3:** Baseline characteristics prior to PRB initiation

	**Group 1 (*n* = 5)**	**Group 2 (*n* = 10)**
Median age, years (range)	37 (34–45)	36 (27–47)
Sex, n (%)		
Male	4 (80)	1 (10)
Female	1 (20)	9 (90)
Baseline OAT, *n* (%)		
Methadone (≤ 30 mg)^a^	1 (20)	1 (10)
Oral buprenorphine lyophilisate	4 (80)	0 (0)
None (out of treatment)	-	9 (90)
UDS, n (%)		
Buprenorphine	4 (80)	2 (20)
Methadone	1 (20)	4 (40)
Cocaine	0 (0)	9 (90)
Morphine	0 (0)	10 (100)
Benzodiazapine	0 (0)	6 (60)
Amphetamine	0 (0)	2 (20)
CGI-S score, mean (± SD)	2.6 (± 0.9)	6.4 (± 1.0)
SSQ score, mean (± SD)	21.8 (± 1.9)	5.6 (± 4.9)
CORE-10 score, mean (± SD)	5.2 (± 3.3)	28.9 (± 4.2)

^a^Participants who were stable on > 30 mg methadone were excluded due to the complexity and time needed to switch from high-dose methadone to PRB

### Primary outcomes

#### Feasibility: collection of psychosocial, clinical severity and drug use data

At baseline, physicians were able to complete the CORE-10 assessment, the SSQ and the CGI-S scale for all participants across both groups. In addition, all participants provided a sample for a baseline UDS. At the 6-month follow-up, all of group 1 completed the CGI-S, CORE-10 and SSQ assessments. One participant was unable to provide a UDS due to COVID-19 restrictions. In group 2, three participants dropped out of treatment and contact prior to the 6-month follow-up and no follow-up data could be collected. Of the seven participants remaining in treatment, CGI-S scale data were collected for all seven (100%), while 6/7 (86%) completed the SSQ and CORE-10 assessments. 5/7 (71%) provided a follow-up UDS; the remaining two participants were unable to provide samples due to COVID-19 restrictions.

#### Acceptability: treatment adherence and treatment preference

At the 6-month follow-up, all participants in group 1 and 7/10 (70%) in group 2 remained on treatment and had received all doses of PRB, indicating full adherence to the treatment regimen. In group 2, 3/10 (30%) participants withdrew from treatment early at 1, 2 and 3 months post-PRB initiation. Across all 10 participants in group 2, the mean percentage of days spent on treatment across the 6-month period was 80% (SD, 33%; min–max range, 17–100%).

At 6-month follow-up, all participants remaining in the study across both groups opted to remain on monthly PRB therapy rather than return to daily OAT. The three participants from group 2 who discontinued treatment early could not be contacted for feedback on treatment preference.

### Secondary outcomes

#### On-top drug use

At baseline, all participants in group 1 tested negative for on-top drug use by UDS and remained negative for on-top drug use at 6-month follow-up, as assessed by either UDS (4/5 participants) or self-reporting if a UDS could not be completed due to COVID-19 restrictions (1/5 participants). In group 2, baseline UDS results detected morphine in all participants and cocaine in 9/10 (90%) participants. Of those remaining on treatment at 6-month follow-up, 57% tested negative for on-top drug use by UDS. Of the three participants in whom on-top drug use was recorded, one had a positive UDS for cocaine and morphine, one self-reported use of cocaine and heroin, and one self-reported use of heroin (Table 4).

**Table 4:** Follow-up data: 6 months post-PRB initiation

	Group 1 (*n* = 5^*^)	Group 2 (*n* = 7*)
UDS or self-reported, *n* (%)		
Buprenorphine	5 (100)	7 (100)
Methadone	0 (0)	0 (0)
Cocaine	0 (0)	2 (29)
Morphine/heroin	0 (0)	3 (43)
Benzodiazepine	0 (0)	0 (0)
Amphetamine	0 (0)	0 (0)
Negative for on-top drug use, *n* (%)	5 (100)	4 (57)

^*^UDS was not performed in one participant from group 1 and two participants from group 2 due to COVID-19 restrictions. Where a sample for UDS could not be collected, self-reported drug use was recorded instead. Follow-up data could not be collected for a further three participants from group 2, who withdrew from the study early

#### Psychosocial and clinical severity measures

**Fig. 1:**
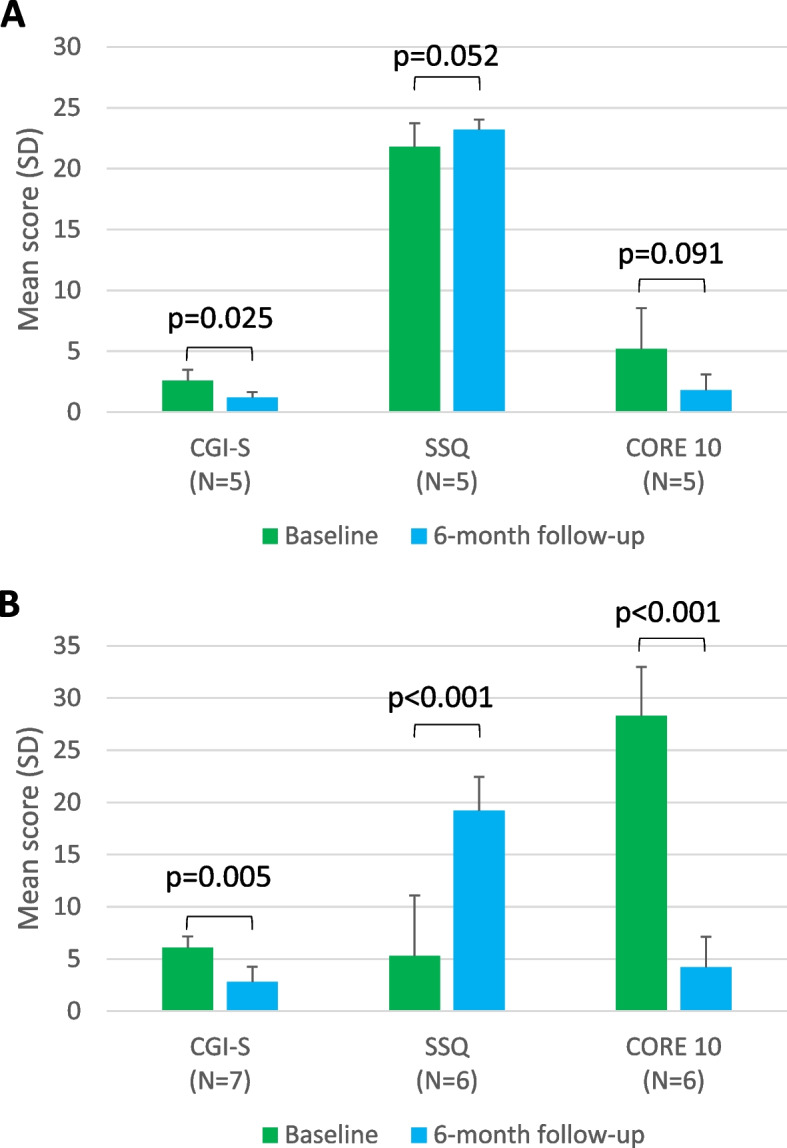
Psychosocial and clinical severity scores at baseline and 6-month follow-up. Mean scores for psychosocial and clinical severity assessments are shown for group 1 (**A**) and group 2 (**B**). CGI-S measures the clinical severity of a participant’s illness, with a lower score indicating less severe disease. SSQ measures the degree of available social support and satisfaction with the social environment for each participant, with a higher score indicating higher levels of social support/satisfaction. CORE-10 measures levels of psychological distress, with a lower score indicating less distress. In group 2, three participants withdrew from treatment before 6-month follow-up and scores could not be collected. One further participant failed to complete the follow-up CORE-10 and SSQ assessment despite remaining on treatment, so was excluded from this analysis. Data shown are mean ± SD. Two-tailed, paired sample *t*-tests were used to determine statistical differences

Marked psychosocial and clinical severity improvements were evident in both groups at follow-up compared with baseline (Fig. 1). Between baseline and 6 months, mean CGI-S scores decreased significantly from 2.6 to 1.2 (*p* = 0.025) for group 1, and from 6.1 to 2.8 (*p* = 0.005) for group 2. Mean SSQ scores increased from 21.8 to 23.2 (*p* = 0.052) for group 1 and from 5.3 to 19.2 (*p* < 0.001) for group 2. In group 2, CORE-10 scores decreased significantly from 28.3 to 4.2 (*p* < 0.001) between baseline and 6 months. In group 1, there was a trend towards a reduction in CORE-10 scores at 6 months post-PRB initiation. Psychosocial improvements indicated by decreased CGI-S and CORE-10 scores and increased SSQ scores were particularly marked among those in group 2 (Fig. 1).

#### Treatment response

**Fig. 2:**
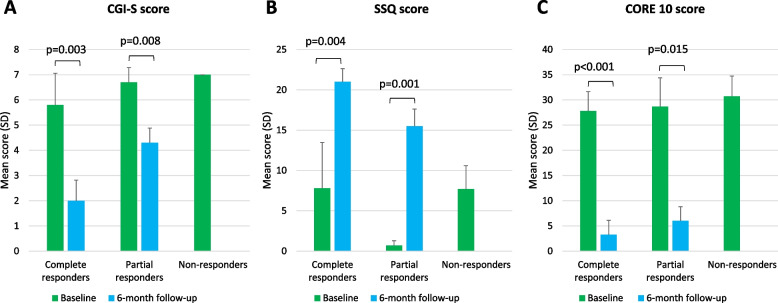
Psychosocial and clinical severity scores among participants in group 2 by treatment response category. CGI-S scores (**A**), SSQ scores (**B**) and CORE-10 scores (**C**) were collected for all members of group 2 at baseline (*N* = 10). Four participants were classified as complete responders, three as partial responders and three as non-responders. Follow-up scores could not be collected for the three non-responders. In addition, one partial responder did not provide follow-up data for the SSQ and CORE 10 scores. Data are mean ± SD. Two-tailed *t*-tests were used to determine statistical differences

In group 1, all participants were classed as complete responders. Treatment response in group 2 was more varied, with 4/10 (40%) participants showing a complete response, with full adherence to treatment and no detected or reported on-top drug use, and three (30%) showing a partial response, with full treatment adherence but continued use of heroin and/or cocaine. The final three participants were non-responders who discontinued treatment in ≤ 3 months. Of note, group 2 participants showed marked improvements in CGI-S, CORE-10 and SSQ scores regardless of whether they were complete or partial responders (Fig. 2).

#### Return to employment or education

In group 1, 3/5 participants were in stable employment at baseline and remained employed at 6-month follow-up. At baseline, no participants from group 2 were engaged in education or employment. Of the seven participants remaining in treatment at 6-month follow-up, one had returned to employment and two had returned to college education. No employment or education data are available for the three participants who discontinued treatment prior to 6-month follow-up.

## Discussion

This pilot study investigated the feasibility, acceptability, and efficacy of PRB therapy in OUD from two different groups: those already adhering well to daily OAT (group 1), and those not currently adhering to or responding to daily OAT (group 2). Across both groups, the treatment protocol was shown to be feasible and acceptable. High treatment adherence rates indicate that attending a clinic once monthly was achievable for all those in group 1 and for the majority in group 2. Data collection was also shown to be feasible, with data on clinical severity and psychosocial measures successfully collected for all participants from group 1 and the majority from group 2. Due to the presence of COVID-19 restrictions at the time, it was not possible to collect UDS samples for all participants. As an alternative, participants were willing to self-report drug use where a UDS could not be performed.

All participants who adhered to PRB therapy throughout the study opted to remain on PRB after the 6-month follow-up, rather than switch back to daily OAT, demonstrating high levels of acceptability. This is in line with other studies on treatment satisfaction with PRB therapy. In one randomised clinical trial comparing daily sublingual buprenorphine with PRB, the latter was associated with greater treatment satisfaction, lower treatment burden, increased quality of life and improved physical functioning [13]. An Australian study designed to explore the practical and social implications of PRB therapy from a patient perspective reported benefits such as opportunities to avoid stigma at pharmacy and clinic visits, more time to engage in daily activities like work and travel and cost savings by eliminating the pharmacy fees associated with daily dosing [26]. In contrast, some patients felt that PRB therapy reduced their ability to engage with important social and practical supports available at pharmacies and clinics [26].

This pilot study also demonstrated the potential for high levels of efficacy in the treatment of both groups. Psychosocial and clinical assessment scores improved for all those who remained in treatment at 6 months. It was noteworthy that in group 2, a population with a history of non-response to daily OAT, 70% of participants showed a complete or partial response to PRB treatment. Furthermore, all complete or partial responders from group 2 demonstrated marked improvements in psychosocial and clinical scores, indicating they experienced improvements in their quality of life regardless of whether they were able to completely abstain from on-top drug use. This pilot study therefore supports the use of PRB therapy as a viable treatment option which should be considered for patients unable to adhere to daily OAT, as well as treatment-adherent patients. This contrasts with previous narratives surrounding use of PRB, in which patients on stable therapy were considered most likely to benefit [21].

It was not possible to contact three participants from group 2 who discontinued treatment to assess key outcomes, treatment preferences or drug use. It should be noted that, while these participants all displayed high levels of clinical severity at baseline (CGI-S score = 7), there were no clear baseline characteristics that may have predisposed them to treatment discontinuation compared with other participants in group 2. A larger study may be able to provide more insight into factors associated with treatment discontinuation. Of note, 9/10 participants in group 2 were female, including the three who discontinued treatment, compared with 1/5 in group 1. Whilst recruitment into future studies should be assessed based on clinical need and not sex, analysis of sex differences between groups and outcomes may provide valuable insights into the needs of women with OUD.

The higher costs associated with PRB therapy also necessitate a thorough appreciation of which patients would benefit the most from treatment. In particular, patients who cannot adhere to daily OAT are associated with relatively high direct and indirect healthcare costs. This pilot study indicates that an evaluation of the cost-effectiveness of PRB treatment over daily OAT in this population would be warranted [4].

Of note, this pilot study utilised an induction protocol which aimed to ensure the rapid transition of participants onto PRB. Previous studies have demonstrated that risk of opioid withdrawal onset, treatment discontinuation and relapse is increased in patients receiving subtherapeutic doses of OAT and that rapid transition between therapies is essential to reduce this risk [27–30]. Due to the complexity and risk associated with transitioning patients from higher dose (> 30 mg) methadone onto PRB, these patients were excluded from this pilot study. Future trial protocols must consider whether these patients should be included and, if so, how to transition participants onto PRB without increasing risk of opioid withdrawal and relapse. Recent papers have described the use of buprenorphine micro-dosing as one possible method to achieve rapid transition for patients taking > 30 mg methadone [31–33].

The main limitation of this study was the small sample size from a single clinic location. It is recommended that future trials recruit from across a range of locations to obtain a diverse sample. There was no active comparator group receiving daily supervised therapy. The study was not powered to detect statistical differences, and the statistics shown in the paper are indicative and not pre-specified. The CORE-10 assessment has not been well validated in the field of substance use disorders and a larger subsequent trial may benefit from an alternative assessment, such as the Generalised Anxiety Disorder (GAD7) questionnaire or the Patient Health Questionnaire (PHQ-9). The use of semi-structured interviews was effective for self-reporting of drug use and suited to a pilot study format. However, they did not produce quantifiable data on participant opinions and perspectives. Future trials may consider the use of questionnaires at baseline and follow-up appointments to increase understanding of patient perspectives regarding PRB therapy and identify factors contributing to treatment success or failure.

## Conclusions

The study design was found to be feasible and the use of PRB therapy was acceptable to participants as an alternative to daily OAT. Adherence to the monthly treatment regimen was high for both groups, and all those remaining on PRB at 6-month follow-up opted to persist with PRB therapy. Potential efficacy was positive for improvements in psychosocial assessment scores, clinical severity measures and reduced on-top drug use, with a particularly marked improvement across these measures observed for group 2 participants, who could not previously adhere to daily OAT. A larger randomised controlled trial is warranted.

## Data Availability

The datasets used and/or analysed during the current study are available from the corresponding author on reasonable request.

## Abbreviations

CGI-S: Clinical Global Impression Severity scale
CONSORT: CONsolidated Standards Of Reporting Trials
CORE-10: Clinical Outcome Routine Evaluation 10
COVID-19: Coronavirus disease 2019
GAD7: Generalised Anxiety Disorder questionnaire
ICD: International Classification of Diseases
NICE: National Institute for Clinical Excellence
OAT: Opioid agonist therapy
OOT: Out-of-treatment
OUD: Opioid-use disorder
PHQ-9: Patient Health Questionnaire-9
PRB: Prolonged-release buprenorphine
SSQ: Social Satisfaction Questionnaire
UDS: Urine drug screen
